# Purinergic Signalling in Parkinson’s Disease: A Multi-target System to Combat Neurodegeneration

**DOI:** 10.1007/s11064-019-02798-1

**Published:** 2019-05-04

**Authors:** Adrián Tóth, Zsófia Antal, Dániel Bereczki, Beáta Sperlágh

**Affiliations:** 1 Department of Neurology, Faculty of Medicine, Semmelweis University, Balassa u. 6., Budapest, 1083 Hungary; 2 Institute of Experimental Medicine, Hungarian Academy of Sciences, Szigony u. 43., Budapest, 1083 Hungary; 3 János Szentágothai School of Neurosciences, Semmelweis University School of PhD Studies, Üllői út 26., Budapest, 1085 Hungary

**Keywords:** Adenosine, Adenosine receptors, ATP, Parkinson’s disease, Purinergic receptors

## Abstract

Parkinson’s disease (PD) is the second most common neurodegenerative disorder, characterized by progressive loss of dopaminergic neurons that results in characteristic motor and non-motor symptoms. l-3,4 dihydroxyphenylalanine (l-DOPA) is the gold standard therapy for the treatment of PD. However, long-term use of l-DOPA leads to side effects such as dyskinesias and motor fluctuation. Since purines have neurotransmitter and co-transmitter properties, the function of the purinergic system has been thoroughly studied in the nervous system. Adenosine and adenosine 5′-triphosphate (ATP) are modulators of dopaminergic neurotransmission, neuroinflammatory processes, oxidative stress, excitotoxicity and cell death via purinergic receptor subtypes. Aberrant purinergic receptor signalling can be either the cause or the result of numerous pathological conditions, including neurodegenerative disorders. Many data confirm the involvement of purinergic signalling pathways in PD. Modulation of purinergic receptor subtypes, the activity of ectonucleotidases and ATP transporters could be beneficial in the treatment of PD. We give a brief summary of the background of purinergic signalling focusing on its roles in PD. Possible targets for pharmacological treatment are highlighted.

## Introduction

### Parkinsons’s Disease: Pathophysiological Background

Parkinson’s disease (PD) is the second most common neurodegenerative disorder, characterized by progressive loss of dopaminergic neurons in the *substantia nigra pars compacta* that results in dopamine (DA) deficiency in the striatum. The ongoing degeneration of this peculiar pathway causes the characteristic motor symptoms such as resting tremor, rigidity, bradykinesia and postural instability [1, 2]. Besides dopaminergic neural degeneration, the presence of Lewy bodies (protein aggregates) due to misfolding of α-synuclein occurs in various regions of the affected brain [3]. In spite of many studies on the pathogenesis of PD, the precise mechanism underlying these events has not been unraveled yet. However, a genetic predisposition associated with disturbed proteostasis due to impaired ubiquitin–proteasome system, mitochondrial dysfunction, oxidative stress and neuroinflammation seems to play cardinal roles for the α-synuclein aggregation and the progression of pathology in PD [4–7]. Among these factors, the pathological, self-amplifying interaction between mitochondrial dysfunction and oxidative stress has been early recognized, which might be a key factor responsible for the selective vulnerability of dopaminergic neurons in PD, and one potential reason behind the clinical failures of neuroprotective therapies so far [8]. Dysfunction of the mitochondrial complex I results in an enhanced production of reactive oxygen species (ROS), which, in turn will inhibit complex I and other vital metabolic enzymes such as alpha-ketoglutarate dehydrogenase, whilst the latter also serves as a source of ROS generation in mitochondria [9, 10]. Simultaneous or preceding mitochondrial dysfunction exacerbates the effect of oxidative stress on pathological monoamine release from nerve terminals [11, 12]. This process leads to the formation of toxic, oxidative DA metabolites, such as dopamine quinone, which might further amplify the ongoing degeneration process [13]. Therefore, disease-modifying potential could be primarily expected from those novel multi-target therapies, which simultaneously target the above mentioned pivotal pathological pathways and prevent their pathological interaction [14, 15].

### The Current Treatment of PD

As for the symptomatic treatment of PD, the clinical break-through came with the first clinical trials of DA replacement therapy using the high dosage of the DA precursor l-3,4 dihydroxyphenylalanine (l-DOPA) [16–19]. l-DOPA is able to cross the blood–brain barrier and converts into DA that engages specific DA receptor subtypes (D_1_ to D_5_) [20]. However, long-term use of l-DOPA leads to a dysbalance of striatal circuits of the motor system and leads to side effects such as l-DOPA induced dyskinesias and motor fluctuation in 50% of patients after 5 years of continuous treatment [21, 22]. The therapeutic management of these complications is difficult and there is a need for developing effective and new pharmacological therapies against motor fluctuation and dyskinesias [23].

### Purinergic Signalling: Concept and Purinergic Receptors

The concept of purinergic signalling, being adenosine 5′-triphosphate (ATP) as an extracellular signalling molecule with neurotransmitter properties was proposed in the early 1970s [24, 25]. A couple of years later, purines were also described as co-transmitters and neuromodulators in the peripheral and central nervous system (CNS), as they are able to modulate other signalling pathways and neurotransmitter systems [26–28]. ATP is co-released with acetylcholine, catecholamines, γ-amino butyric acid (GABA), glutamate and DA in the CNS [29–34]. Extracellular ATP is released from cells under physiological conditions. The levels of extracellular ATP are controlled by ectonucleotidases that catalyze its degradation [35, 36].

There are two families of purinergic receptors, which are distinguished by their main agonists [37]. P1 receptors are G protein-coupled metabotropic receptors activated by adenosine and can be subdivided into four subtypes (A_1_, A_2A_, A_2B_, A_3_). P2 receptors are subdivided into two classes: P2X_(1-7)_ ionotropic receptors, activated by ATP and G protein-coupled metabotropic P2Y_(1-2,4,6,11-14)_ receptors, activated by ATP, adenosine diphosphate (ADP), uridine di- and triphosphate (UDP and UTP), or UDP-glucose depending on the receptor subtype [38–40]. ATP is able to bind to the extracellular ligand-binding site of P2X receptors and leading to conformational change that opens a permeable channel to Na^+^, K^+^ and Ca^2+^. The activation of these ionotropic receptors is important for Ca^2+^-induced intracellular signalling pathways [41–43]. Depending on the activated adenosine and P2 receptor subtype, the induced signalling pathway may vary. These activated receptors are able to make alterations in Ca^2+^ levels, which modulate the activity of several secondary messengers involved in physiological processes [44–46]. The final effects of purinergic receptor-mediated signalling depend on the cell type and other physiological (neurogenesis, proliferation, cell death, stem cell differentiation) or pathological cellular conditions (inflammatory, neurological, psychiatric, oncological, cognitive, neuromuscular and neuromotor diseases) [47–66]. Purinergic receptor activation may have para- or autocrine nature, which is characteristic for astrocytes in the regulation of neuronal activity [67]. Not only purinergic receptors but membrane nucleotide/nucleoside transporters, channels and ectonucleotidases also play important role in purinergic signalling [36, 68–70].

Adenosine is the predominant, presynaptic modulator of neurotransmitter release in the CNS, although ATP has presynaptic modulator effect as well [71–73]. Adenosine is produced by enzymatic breakdown of released ATP, but some CNS cells are able to release adenosine directly [74]. A_1_ and A_2A_ receptors have higher affinity (activated by physiological extracellular levels of adenosine) and A_2B_ and A_3_ receptors have lower affinity (activated by higher extracellular levels of adenosine) for the ribonucleoside [75–77]. The adenosine A_1_ and A_2A_ receptors are highly expressed in the brain and CNS, where they have profound influence on neuronal activity. Adenosine A_1_ receptor is the dominant adenosine receptor subtype in the CNS. Adenosine A_1_ receptors can be found in various cortical and subcortical regions of the brain, while A_2A_ receptors are mainly expressed in the striatum [78–81] (Table 1). In contrast, adenosine A_2B_ and A_3_ receptors are mainly found in peripheral tissues, even though low levels of these receptors are also expressed in some regions of the brain [82–84].

**Table 1:** Localization of adenosine receptor subtypes in CNS [80, 81]

	CNS
A_1_	High levels in striatum, thalamus and moderate levels in cortex, pons
A_2A_	High levels in striatum, thalamus, hippocampus
A_2B_	Low levels in microglia cells, astrocytes
A_3_	Low levels in cortex, hippocampus, striatum, cerebellum

There is a heterogeneous distribution of P2 purinergic receptors in the CNS as well. For instance P2X_1_ receptors are predominantly expressed in the cerebellum, while P2X_3_ receptors are expressed in the brainstem [85, 86], and they can be found in the basal ganglia with variable expression level [87] (Table 2). Various P1 and P2 receptor subtypes are also expressed by microglia, astrocytes and oligodendrocytes [88–93]. Extracellular nucleotides act as messengers between neuronal and non-neuronal cells, thereby integrating functional activity between neurons, glial and vascular cells in the CNS [94–98]. Adenosine and ATP—as key players in neuron–glia interaction and microglial activation—are modulators of neuroinflammatory processes, oxidative stress, excitotoxicity and cell death [99–102]. Aberrant purinergic receptor signalling can be the cause or result of numerous pathological conditions, including neurodegenerative disorders [103]. Here, we explore the importance of purinergic signalling in PD to suggest potential targets for novel therapies.

**Table 2:** Expression of P2 receptor subtypes in the basal ganglia (striatum and substantia nigra) [87]

	Striatum	Substantia nigra
P2X_1_	↑↑↑	↑↑↑
P2X_2_	↑↑↑	↑↑↑
P2X_3_	↑↑	↑↑
P2X_4_	↑↑↑	↑↑↑
P2X_5_	↑	↑↑↑
P2X_6_	↑	↑↑
P2X_7_	↑↑	↑↑
P2Y_1_	↑	↑↑
P2Y_2_	↑↑↑	↑↑↑
P2Y_4_	↑↑↑	↑↑↑
P2Y_6_	↑	↑↑↑
P2Y_11_	–	–
P2Y_12_	↑↑↑	↑↑↑
P2Y_13_	–	–
P2Y_14_	–	↑↑↑

Expression level of P2 receptor subtypes: – = no expression, ↑ = low expression, ↑↑ = medium expression, ↑↑↑ = high expression

## Purinergic Signalling Involvement in PD

### Purinergic Gene Polymorphisms in PD

Two *ADORA2A* (A_2A_ receptor) polymorphisms (rs71651683, a 5′ variant or rs5996696, a promoter region variant) were inversely associated with genetic PD risk, moreover, there was evidence of interaction with coffee consumption [104]. CYP1A2a is an enzyme, which is responsible for caffeine metabolism, two polymorphisms (rs762551 or rs5996696) of the enzyme in homozygous coffee drinkers reduced PD risk [104]. Humans with R1628P variant (LRRK2 risk variant) who did not take caffeine had a 15 times increased risk of PD [105]. *GRIN2A* encodes an *N*-methyl-d-aspartate-2A (NMDA) glutamate receptor subunit involved in central excitatory neurotransmission, which is associated with A_2A_ receptor activation. Carriers of *GRIN2A* rs4998386-T allele had a lower risk of PD, than carriers of rs4998386-CC genotype among heavy coffee drinkers [106]. There is evidence that creatine is able to hasten PD progression in *GRIN2A* coffee drinkers, which demonstrates an example of a genetic factor interacting with environmental factors exemplifying the complexity of environment–gene interactions in the progression of PD [107]. In addition, P2X_7_ receptor 1513A>C polymorphism is a risk factor for sporadic PD, late-onset PD and male PD in Han Chinese population [108].

### Adenosine Receptor-Mediated Signalling in PD

A_2A_ receptors are enriched in dopaminergic brain areas (the highest expression of these receptors are in the striatum), thus pointing to a significant role of purines in motor control [109]. A_2A_ and DA D_2_ receptors are mainly expressed in the neurons of the indirect pathway of striatal circuits projecting to the *globus pallidus*, in contrast to A_1_ and DA D_1_ receptors, which are mainly found on the neurons of the direct pathway of motor control projecting to the internal *globus pallidus* and *substantia nigra pars reticulata*. The main adenosine signalling mechanism is via the cyclic adenosine monophosphate (cAMP)-dependent pathway. Activated A_2A_ receptors stimulate the enzymatic function of adenyl cyclase that increases cAMP levels and depresses the signalling mediated by D_2_ receptors. Activation of protein Gi-coupled DA D_2_ receptors leads to reduction in the cAMP level. There is a reciprocal situation in the direct pathway of motor control with protein Gs-coupled D_1_ and protein Gi/o-coupled A_1_ receptors. Generally, adenosine acts as a negative modulator of D_1_- and D_2_-mediated actions in the direct and indirect pathways [110–112].

The antagonistic functional interaction between adenosine A_2A_ and DA D_2_ receptors may depend on the formation of receptor heterodimers (A_2A_-D_2_ heteroreceptor complexes) in the striatum thereby balancing the inhibitory and excitatory impulses in the striatal circuits [112]. Not only dopaminergic mechanisms, but non-dopaminergic modes of action of A_2A_ receptors may involve interactions with various non-dopaminergic receptors, possibly by forming heterodimeric and/or multimeric receptor complexes [23]. Thus, adenosine A_2A_ receptors may adjust the actions of striatal adenosine A_1_ receptors (A_1_-A_2A_ heteroreceptor complexes), metabotropic glutamate receptors (mGlu) 5 (A_2A_-mGlu_5_ heteroreceptor complexes), cannabinoid receptor type 1 (CB_1_) receptors (A_2A_-CB_1_ heteroreceptor complexes) and serotonin 1A (5-HT_1A_) receptors [113–115]. Moreover, studies also suggested the presence of multimeric A_2A_-D_2_-mGlu_5_ and A_2A_-CB_1_-D_2_ receptor complexes in the striatum [116, 117]. These functional interactions between receptors may modulate the activity of striatal efferent neurons and influence motor behavior [23]. In general, adenosine tone appears as a key for the fine tune control of DA dependent actions in the basal ganglia and affects non-dopaminergic mechanisms also [20].

Adenosine receptor antagonists (especially non-selective A_2A_ receptor antagonists, such as methylxanthines, caffeine, or selective A_2A_ antagonists) have been shown to enhance therapeutic effect of l-DOPA in a wide range of animal models of PD [118–121]. A_2A_ homoreceptor complexes are in balance with DA D_2_ homoreceptor complexes in intact striatum [122–126]. Dysbalance of striatal circuits leads to motor inhibition and disruption of this balance in PD leads to increased signalling via A_2A_ receptors and decreased signalling via DA D_2_ receptors. These changes explain the beneficial effect of A_2A_ receptor antagonists on increasing motor functions without worsening l-DOPA-induced dyskinesias [20, 127].

A_2A_ receptor antagonists have been used in clinical trials in patients with PD (Table 3). Istradefylline is a xanthine-based compound with increased selectivity for A_2A_ receptors against A_1_ receptors, which is used concomitantly with l-DOPA [128]. The drug was not approved in the USA because there was no significant reduction in *off time* compared to l-DOPA treatment [129]. In contrast, istradefylline was approved in Japan in 2013 with the trade name Nouriast^®^ to enhance the antiparkinsonian effect of l-DOPA with less long-term side effects [130, 131]. Preladenant is a second-generation A_2A_ receptor antagonist, which failed in phase III clinical trials in the treatment of PD because the compound was not superior to placebo in reducing *off state* [132, 133]. Vipadenant is a triazolopyromidine-based drug, which has increased selectivity for A_2A_ receptors versus A_1_ and A_3_ receptors [134]. Its development as an antiparkinsonian medication was stopped; however, A_2A_ receptor antagonists have considerable potential in novel immune-oncology and cardiology therapies [113, 135–137]. Another adenosine A_2A_ receptor antagonist, tozadenant was safe, well tolerated and effective in reducing *off time* in PD patients in phase II trial but phase III clinical trial was discontinued because of serious adverse events (agranulocytosis) [23, 133, 138]. There have been many drug trials for selective A_2A_ receptor antagonists. Most of them were shown to be safe, well tolerated and beneficial; however, the majority did not reach the regulatory threshold for efficacy to be approved as PD drugs [139, 140]. Development of bivalent drugs (able to bind to two receptors simultaneously) to target A_2A_-D_2_ heteroreceptor complexes acting on A_2A_ and DA D_2_ receptors may be a good therapeutic approach in the future. Heterobivalent drugs offers the opportunity to target the orthosteric sites of the receptors in the heterodimer with a higher affinity and a higher specificity versus corresponding homomers and reduce the dose required for therapy and, accordingly, the side effects [20].

**Table 3:** Pre-clinical and clinical studies with purinergic receptor antagonists in PD

Compounds	Mechanism of effect	Models	Published	Results
KW-6002 (istradefylline)	A_2A_ receptor antagonism	PD patients	2003	Improved PD motor scores when added to low-dose l-DOPA
KW-6002 (istradefylline)	A_2A_ receptor antagonism	LPS treated rats	2013	Enhanced therapeutic effect of l-DOPA
Caffeine	A_2A_ receptor antagonism	LPS treated rats	2013	Reduced motor impairment
Preladenant	A_2A_ receptor antagonism	MPTP treated mice	2014	Enhanced therapeutic effect of low doses of l-DOPA
8-Ethoxy-9-ethyladenine	A_2A_ receptor antagonism	6-OHDA lesioned rats	2015	Enhanced effect of low doses of l-DOPA without increased dyskinesia
SCH 58261	A_2A_ receptor antagonism	A_2A_ receptor knockout mice, SH-SY5Y cells	2015	Decreased α-synuclein aggregation, prevented neuronal death
ZM 241385	A_2A_ receptor antagonism	A_2A_ receptor knockout mice, SH-SY5Y cells	2015	Decreased α-synuclein aggregation, prevented neuronal death
Preladenant	A_2A_ receptor antagonism	PD patients	2017	Failed (was not superior to placebo) in phase III clinical trial
Vipadenant	A_2A_ receptor antagonism	PD patients	2009	Failed (was not superior to placebo)
Tozadenant	A_2A_ receptor antagonism	PD patients	2017	Failed in phase III clinical trial (induced agranulocytosis)
NF449	P2X_1_ receptor antagonism	H4 cells	2015	Prevented α-synuclein aggregation
A-438079	P2X_7_ receptor antagonism	6-OHDA lesioned rats	2010	Prevented depletion of DA in striatum
BBG	P2X_7_ receptor antagonism	6-OHDA lesioned rats	2014	Reverted dopaminergic neurons loss in substantia nigra
BBG	P2X_7_ receptor antagonism	BV2 microglia cells	2015	Decreased ROS production induced by α-synuclein
PPADS	P2X_7_ receptor antagonism	SH-SY5Y cells	2017	Prevented abnormal calcium influx induced by α-synuclein
AZ 11645373	P2X_7_ receptor antagonism	SH-SY5Y cells	2017	Prevented abnormal calcium influx induced by α-synuclein
AP4A	P2Y_2_/P2Y_4_ antagonism	6-OHDA lesioned rats	2003	Reduced dopaminergic neurons loss
MRS2578	P2Y_6_ receptor antagonism	SH-SY5Y cells	2017	Delayed neuronal loss

The list is not comprehensive and is restricted to studies mentioned in the article. For further references, see [111, 113]

Adenosine A_2A_ receptor antagonists may also involve direct or indirect actions at microglia and inflammatory processes. Pre-treatment of slices from 1-methyl-4-phenyl-1,2,3,6-tetrahydropyridine (MPTP)-injected mice with preladenant facilitates the ability of activated microglia to respond to tissue damage [141]. The nonselective A_1_/A_2A_ adenosine receptor antagonist caffeine and the selective A_2A_ receptor antagonist (KW-6002) had anti-inflammatory potential in a rat model of lipopolysaccharide (LPS)-induced neuroinflammation [142].

### The Role of A_2A_ Receptors in Synucleopathy

Increased striatal A_2A_ receptor expression was observed as an early pathological event in PD and increased A_2A_ receptor expression was detected after hippocampal injection of α-synuclein in mice [143, 144]. A_2A_ receptor-knock out mice showed resistance against α-synuclein induced insults [145]. A_2A_ receptor antagonism restrained hyperactivation of NMDA-glutamate receptors and decreased the aggregation of α-synucleins [146]. Based upon these results, A_2A_ receptors seem to have role in the pathological process of synucleinopathy [111].

### P2 Receptor-Mediated Signalling in PD

P2 ionotropic and metabotropic receptors are widely expressed in basal ganglia and in various cell types, such as neurons and astrocytes [87, 147, 148]. 6-Hydroxidopamine (6-OHDA) induced lesions of nigral dopaminergic neurons generate a significant decrease in the expression of P2X and P2Y receptor proteins from striatal spiny neurons and GABAergic interneurons, thus confirming the involvement of P2 receptors and extracellular ATP in the striatal circuits [87]. P2Y_1_ and P2X_1-4, 6_ receptor protein subtypes are expressed in dopaminergic neurons with co-expression of P2X_1_ with DA D_1_ receptors, therefore stimulation of P2 receptors by ATP induces an increased release of DA in the striatum [149–152]. In a neuronal cell model, extracellular ATP induced a significant increase in intracellular α-synuclein levels, which was the result of lysosome dysfunction caused by P2X_1_ receptor activation [153].

Many data have implicated the role of P2X_7_ receptor in PD. P2X_7_ receptor antagonism with A-438059 or Brilliant Blue G (BBG) prevented DA deficit in the striatum and 6-OHDA-induced hemiparkinsonian behavior [154, 155]. However, P2X_7_ receptor deficiency or inhibition did not promote the survival of dopaminergic neurons in rotenone and MPTP induced animal models of PD [156]. It is presumed that there is a massive release of ATP during cell death in the lesioned striatum and *substantia nigra*, which activates cell death pathways via purinergic receptors and is able to activate further purinergic subtypes [20]. Permanent purinergic receptor activation and ATP release seem to play a key role in the neuronal death, which exacerbates α-synuclein aggregation in PD [87]. The accumulation of α-synuclein might overwhelm the capacity of intracellular protein-degradation mechanisms and induce neuroinflammation, which creates a positive feedback loop promoting the degeneration of dopaminergic cells [7]. α-Synuclein-induced intracellular free calcium mobilization in neuronal cells depends on the activation of purinergic P2X_7_ receptors. In the same study, activation of P2X_7_ receptors lead to ATP release with the recruitment of the pore forming protein pannexin1, whilst α-synuclein decreased the activity of extracellular ecto-ATPase which is responsible for ATP degradation [157]. Stimulation of the microglial P2X_7_ receptor by extracellular α-synuclein increased oxidative stress, which was prevented with the use of P2X_7_ receptor antagonist [158].

DA neurotransmission has been linked to calcium signalling. There is data that P2Y_1_ receptor is involved in the regulation of calcium signalling [159]. Neurodegeneration induced by 6-OHDA in nigrostriatal dopaminergic neurons was reduced by pretreatment with diadenosine tetraphosphate (AP4A, an endogenous diadenosine polyphosphate) possibly through an anti-apoptotic mechanism and the activation of P2Y_1_ and P2Y_4_ receptors [160]. Recently, expression levels of P2Y_6_ receptor in PD patients younger than 80 years were higher than healthy controls and multiple system atrophy (MSA) patients and P2Y_6_ receptor could thereby be a potential clinical biomarker of PD. P2Y_6_ receptor was also upregulated in LPS-treated microglial cells and involved in proinflammatory cytokine release through UDP secretion [161]. Another study showed that expression of P2Y_6_ receptor on neuronal SH-SY5Y cell is associated with the progression of oxidative stress and cell death induced by 1-methyl-4-phenylpyridinium (MPP^+^) [162]. In vivo, LPS induced microglial activation and delayed neuronal loss was prevented by selective inhibition of P2Y_6_ receptor with MRS2578 [163]. Based on these studies P2Y_6_ receptor subtype seems to be involved in the process of neuroinflammation in PD and blocking UDP/P2Y_6_ receptor signalling could reverse these pathological processes [161].

## Conclusion

In general, many data confirm the involvement of purinergic signalling pathways in PD. Modulation of purinergic receptor subtypes, the activity of ectonucleotidases and ATP transporters could be beneficial in the treatment of PD. Antagonism of A_2A_, P2X_1_, P2X_7_ and P2Y_6_ receptor subtypes is a promising weapon against PD via various ways: reducing l-DOPA induced dyskinesia, influencing neuroinflammation, preventing α-synuclein aggregation, reducing microglia activation. Development of new bivalent compounds to target A_2A_-D_2_ heteroreceptor complexes, which are orally bioavailable and can cross the blood–brain barrier could be a potential therapeutic tool. In addition, multi-target compounds targeting self-amplifying circuits controlled by purinergic and non-purinergic receptors could be a viable strategy to obtain the desired disease-modifying effect [164]. Additional studies and better quality PD animal models are required for the deeper understanding of underlying unknown pathological processes in PD and the role of purinergic signalling in it.

## Abbreviations

ADORA2A: Adenosine A_2A_ receptor
ADP: Adenosine 5′-diphosphate
AP4A: Diadenosine tetraphosphate
ATP: Adenosine 5′-triphosphate
cAMP: Cyclic adenosine monophosphate
CB_1_: Cannabinoid receptor type 1
DA: Dopamine
GABA: γ-Amino butyric acid
GRIN2A: Glutamate ionotropic receptor NMDA type subunit 2A
5-HT_1A_: 5-Hydroxytryptamine/serotonin receptor 1A
l-DOPA: l-3,4 dihydroxyphenylalanine
LPS: Lipopolysaccharide
LRRK2: Leucine-rich repeat kinase 2
6-OHDA: 6-hydroxydopamine
mGlu: Metabotropic glutamate receptor
MPP^+^: 1-Methyl-4-phenylpyridinium
MPTP: 1-Methyl-4-phenyl-1,2,3,6-tetrahydropyridine
MSA: Multiple system atrophy
NMDA: *N*-methyl-d-aspartate
PD: Parkinson’s disease
ROS: Reactive oxygen species
UDP: Uridine 5′-diphosphate
UTP: Uridine 5′-triphosphate
